# Third-Generation Capsule Endoscopy Outperforms Second-Generation Based on the Detectability of Esophageal Varices

**DOI:** 10.1155/2016/9671327

**Published:** 2016-11-17

**Authors:** Sayoko Kunihara, Shiro Oka, Shinji Tanaka, Ichiro Otani, Atsushi Igawa, Yuko Nagaoki, Hiroshi Aikata, Kazuaki Chayama

**Affiliations:** ^1^Department of Gastroenterology and Metabolism, Graduate School of Biomedical Sciences, Hiroshima University, Hiroshima, Japan; ^2^Department of Endoscopy and Medicine, Hiroshima University Hospital, Hiroshima, Japan

## Abstract

*Background and Aim*. The third-generation capsule endoscopy (SB3) was shown to have better image resolution than that of SB2. The aim of this study was to compare SB2 and SB3 regarding detectability of esophageal varices (EVs).* Methods*. Seventy-six consecutive liver cirrhosis patients (42 men; mean age: 67 years) received SB3, and 99 (58 men; mean age, 67 years old) received SB2. All patients underwent esophagogastroduodenoscopy within 1 month prior to capsule endoscopy as gold standard for diagnosis. The diagnosis using SB3 and SB2 for EVs was evaluated regarding form (F0–F3), location (Ls, Lm, and Li), and the red color (RC) sign of EVs.* Results*. SB2 and SB3 did not significantly differ on overall diagnostic rates for EV. Sensitivity, specificity, positive predictive value, and negative predictive value of SB2/SB3 for EV diagnosis were, respectively, 65%/81%, 100%/100%, 100%/100%, and 70%/62%. However, the diagnostic rates for EV form F1 were 81% using SB3 and 52% using SB2 (*P* = 0.009). Further, the diagnostic rates for Ls/Lm varices were 79% using SB3 and 81% using SB2, and, for Li, varices were 84% using SB3 and 52% using SB2 (*P* = 0.02).* Conclusion*. SB3 significantly improved the detectability of EVs compared with SB2.

## 1. Introduction

**Figure 1:**
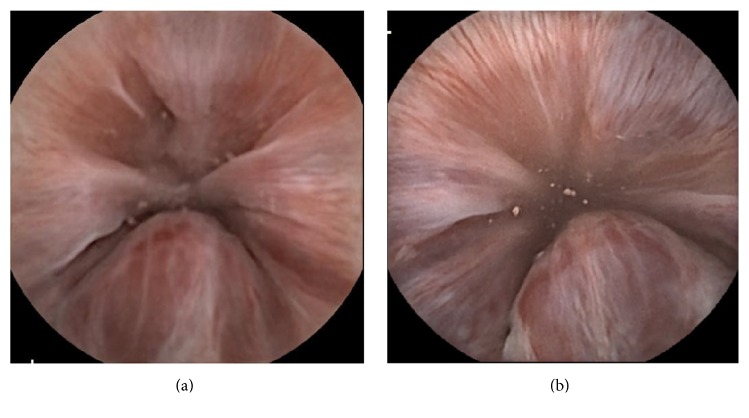
Comparison between second-generation (a) and third-generation (b) capsule endoscopy devices with respect to image resolution. Third-generation devices offer a 30% improvement in image resolution.

Capsule endoscopy (CE) is useful in the diagnosis of small-bowel diseases [1–6]. Recently, the third-generation CE, PillCam® SB3 (SB3), was designed to improve on the second-generation PillCam® SB2 (SB2) in terms of diagnostic confidence and efficiency when assessing and monitoring conditions of the small-bowel. SB2 uses the PillCam recorder “DR2,” and receives two images per second in the small-bowel. On the other hand, SB3 expands the field of vision and automatically adapts the imaging frame rate depending on the speed of capsule passage through the small-bowel. These adaptive frame rate capabilities of SB3 rely on the communication between SB3 and the PillCam recorder “DR3,” which can receive 6 images per second. Indeed, SB3 was found to provide better image resolution (Figure 1).

We previously reported that, compared to double balloon endoscopy (DBE), CE by previous-generation devices (i.e., SB1 and SB2) is characterized by a significant rate of false-negative results when evaluating small-bowel lesions in the proximal jejunum and ileum [7]. While there are several reports about the accuracy of diagnosing small-bowel lesions using SB1 or SB2, no studies have evaluated the diagnostic accuracy of SB3 by being compared to the well-documented accuracy of previous-generation devices. In other words, despite the improved imaging capabilities of SB3, it is not clear whether SB3 is indeed more effective in detecting small-bowel lesions.

The PillCam ESO capsule endoscope is a modified PillCam capsule used for detecting esophageal lesions. There are several reports [2, 8–14] on the usefulness of this capsule for screening the upper gastrointestinal tract in cirrhotic patients, and there has been some speculation that the capsule will make it possible to substitute esophagogastroduodenoscopy (EGD) for esophageal CE. We previously reported that SB2 is effective in the diagnosis of esophageal varices (EVs) and portal hypertensive gastropathy in the gastric body [13]. The present study aimed to assess whether SB3 improves on SB2 in terms of the ability to detect EVs.

## 2. Material and Methods

### 2.1. Patients

This study included 175 consecutive patients (100 men and 75 women; mean age, 67 years; age range, 23–88 years) with liver cirrhosis, who underwent CE at Hiroshima University Hospital between December 2009 and March 2015. Of the 175 patients, 99 (58 men and 41 women; mean age, 67 years; age range, 23–88 years) received SB2 between December 2009 and February 2015 (SB2 group), while 76 patients (42 men; mean age, 67 years; age range, 26–86 years) received SB3 between March 2014 and March 2015 (SB3 group). The indications for CE were suspicion of bleeding from the small-bowel and iron deficiency anemia with a hemoglobin level of <12.0 g/dL. The severity of liver cirrhosis was measured using the Child-Pugh score.

### 2.2. CE Examinations and Findings

CE was performed using SB2 or SB3. The patients swallowed the capsule in the sitting position and were allowed to perform normal activities immediately thereafter. After eight hours, the sensor array and the recording device were removed. Images were analyzed using the Rapid Reader 6.5 software or the RAPID 8 workstation (Given Imaging Ltd., Duluth, GA). Two experienced endoscopists, who were blinded to the EGD findings, evaluated the images captured by CE for the presence or absence of EVs. Diagnosis was established by consensus. The two endoscopists had limited experience with esophageal CE, but they had extensive experience with CE (more than 200 small-bowel examinations) and EGD (more than 3000 examinations).

**Figure 2:**
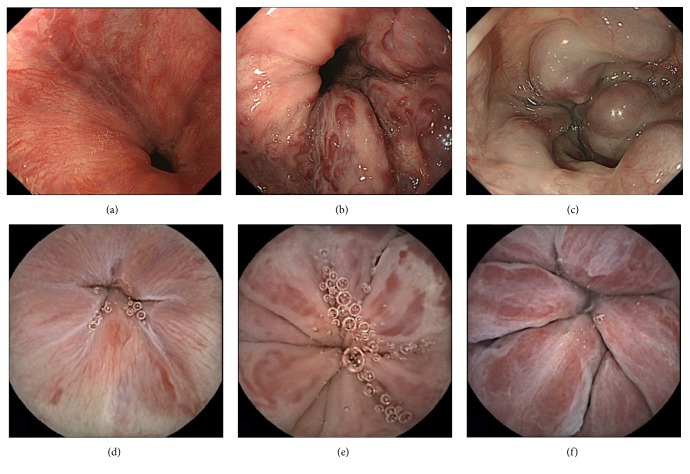
Esophageal varices classified by form, according to their appearance on esophagogastroduodenoscopy. Specifically, (a) straight, small-caliber varices (stage F1); (b) moderately enlarged, beady varices (stage F2); and (c) markedly enlarged, nodular, or tumor-shaped varices (stage F3). The same varices are shown as visualized by capsule endoscopy (d–f), specifically, (d) F1 varices, (e) F2 varices, and (f) F3 varices.

All patients underwent EGD within 1 month prior to CE. On the basis of the EGD findings, which were regarded as the gold standard for diagnosis, the accuracy of CE for specific EV lesions was evaluated. EVs were graded for the EGD procedure according to the general rules for the study of portal hypertension [15]. EVs were described as follows: location (Ls: locus superior; Lm: locus medialis; Li: locus inferior); form (F0: lesion without varicose appearance; F1: straight, small-caliber varices; F2: moderately enlarged, beady varices; F3: markedly enlarged, nodular, or tumor-shaped varices); color (white or blue), and presence of the red color sign (RC sign: red wale marks, cherry red spots, or hematocystic spots). Representative examples of the EVs of various forms, as depicted by EGD and by CE, are shown in Figure 2. Agreement between EGD and CE was assessed using the kappa statistic. Sensitivity, specificity, and positive and negative predictive values were calculated for the use of CE for identification of EVs. In addition, the association between diagnostic yield, form, location, and grade of the varices, as well as between diagnostic rate and esophageal transit time, was evaluated.

The present study was conducted in accordance with the Declaration of Helsinki and was approved by the Institutional Review Board of our hospital. Written informed consent was obtained from all patients who participated in the study.

### 2.3. Statistical Analysis

Data were analyzed by chi-squared test. Yates correction or Fisher's exact test was added when needed. All tests were two-tailed, and comparisons for which *P* < 0.05 were considered statistically significant. All statistical calculations were performed using Microsoft Excel 2008 for Mac (Microsoft, Redmond, WA, USA).

## 3. Results

The clinical characteristics of the liver cirrhosis patients included in our study are shown in Table 1. There were no significant differences between the SB2 and SB3 groups in terms of clinical characteristics. The overall diagnostic yields of CE for EVs, evaluated by comparison against the EGD findings, were 86% and 81% when using SB3 and SB2, respectively, with no significant differences. The diagnostic sensitivity, specificity, positive predictive value, and negative predictive value for SB3 were 81%, 100%, 100%, and 62% respectively, whereas these values were 65%, 100%, 100%, and 70%, respectively, for SB2.

The overall diagnostic rate for F1 EVs was 52% when using SB2 but was significantly higher when using SB3 (81%; *P* < 0.05). On the other hand, the diagnostic rates for F2/F3 EVs were 83% and 81% when using SB2 and SB3, respectively, with no statistically significant difference. Similarly, no significant differences were found between the diagnostic yields of SB2 and SB3 with respect to the location of the detected EVs. Specifically, the diagnostic rates for Ls/Lm EVs were 81% and 79% when using SB2 and SB3, respectively; for Li EVs, the diagnostic rates were 52% and 84% when using SB2 and SB3, respectively (*P* = 0.02). The overall diagnostic rates for positive RC sign were 33% and 65% when using SB2 and SB3, respectively.

The median esophageal transit time was 5 seconds. We found that the diagnostic rates, for shorter transit time (<5 seconds), were 56% and 72% when using SB2 and SB3, respectively, while, for longer transit time (>5 seconds), these were 81% and 79% when using SB2 and SB3, respectively. There was no significant difference between the SB2 and SB3 groups in terms of esophageal transit time (Table 2). However, on average, SB3 allowed to collect a significantly higher number of frames during esophageal passage (33 versus 10; *P* < 0.05). The sensitivity of EV diagnosis was significantly higher for SB3 than for SB2 (81% versus 65%), while the specificity was 100% for both types of capsule, and there were no false-positive cases (Table 3); in the case of straight, small-caliber varices (stage F1), sensitivity was also higher for SB3 than for SB2 (81% versus 52%; Table 4).

## 4. Discussion

According to the guidelines of the European Society of Gastrointestinal Endoscopy [16], CE is currently the first-line imaging modality for diagnosing small-bowel lesions [17–19], especially for patients with obscure gastrointestinal bleeding (OGIB). The reason CE is preferred for such investigations is that the approach is minimally invasive and safe, as the patients can easily swallow the capsule, while the detection capabilities are extensive, and small lesions such as angioectasia of the small-bowel can be diagnosed [20, 21].

We previously reported that total enteroscopy was achieved by both CE and DBE in 54 of 118 patients with OGIB, suggesting a diagnostic yield of 46.3% and 51.9% for CE and DBE, respectively. Moreover, CE was previously shown to have a significant rate of false negatives in detecting small-bowel lesions in the proximal jejunum and ileum [7, 22]. Hadithi et al. [23] reported a diagnostic yield of 80.0% for CE and 60% for DBE; Nakamura et al. [21] reported diagnostic yields of 59.4% and 42.9%, respectively; Ohmiya et al. [24] conducted a multicenter survey of 7 institutions and found yields of 50.0% and 52.7%, respectively. Other reported rates for CE and DBE, respectively, were 71.9% versus 65.6% [25] and 54.1% versus 63.5% [26]. Nakamura et al. [27] reported that SB2 was able to detect the esophageal-cardiac junction, pyloric ring seen from the duodenal bulb, major papilla of the duodenum, ileocecal valve seen from the cecum, vermiform appendix, and anal canal in 17%, 33%, 18%, 20%, 3%, and 2% of cases, respectively; they concluded that detection via SB2 was difficult in the segments of the gastrointestinal tract where SB2 transit time is short. The diagnostic rate is known to depend on the number of images obtained. Because of its adaptive frame rate capabilities, it is expected that SB3 would allow obtaining a higher number of images; indeed, we noted that a significantly higher number of frames were collected during esophageal passage when using SB3 than when using SB2.

Interestingly, we noted significant differences between SB3 and SB2 in terms of diagnostic yield for Li varices but not for Ls/m varices. These findings likely originate from the spatial distribution of varices. Specifically, not all Ls/m varices were isolated to the upper or middle esophagus, and thus both SB2 and SB3 were equally likely to detect them. On the other hand, Li varices were typically located within a narrow area and were thus more easily detectable by SB3, which provides enhanced image acquisition rate in areas where capsule transit is fast.

Overall, our data showed that SB3 was superior to SB2 in terms of diagnostic accuracy for EVs. We thus consider that SB3 improves the detection of small lesions in the small-bowel as it has higher accuracy in detecting F1 EVs, and it decreases the incidence of false-negative observations in the upper jejunum, where CE transmits fast. Monteiro et al. [28] compared the PillCam SB3 and SB2 in terms of the detection rate for the major duodenal papilla, as a surrogate indicator of diagnostic yield in the proximal small-bowel. The PillCam SB3 had a significantly higher detection rate for the major duodenal papilla (42.7% versus 24%; *P* = 0.015), suggesting that SB3 may indeed increase diagnostic yield, particularly in the proximal segments of the small bowel.

Koh et al. [29] reported that the rebleeding rate was 22.8% in OGIB patients with negative CE for more than 6 months. Laine et al. [30] reported that further bleeding rate was 33% in OGIB patients with negative CE for 1 year. Reports regarding patients with OGIB indicated that the rebleeding rate was substantial in patients with negative CE results for more than one year [31]. We propose that SB3 is able to detect sources of bleeding such as small angioectasia, which could not be detected using SB2.

Our study has certain limitations. First, the study included patients from a single center only. Second, it is important to keep in mind that CE does not allow air insufflation, and thus the images are obtained under different physiologic conditions than those present when performing EGD. For this reason, the exact F stage of EVs was difficult to diagnose by CE alone, and staging was performed only on the basis of EGD findings, which were considered as the gold standard. Third, the evaluation was not performed from the small-bowel. Therefore, a large-scale study with evaluation from the upper jejunum (the weakness of SB2-based assessments) is warranted in order to address these limitations.

## 5. Conclusions

Compared to SB2, SB3 showed an improved detection rate for F1 EVs. Therefore, SB3 may improve the diagnostic yield for small-bowel lesions in the upper jejunum, where capsule transit time is short.

## Figures and Tables

**Table 1:** Characteristics of patients with liver cirrhosis (*n* = 175), stratified according to the capsule endoscopy device employed for diagnosis.

Variables	SB2 group	SB3 group	*P* value
	*n* = 99	*n* = 76	
Male/female	58/41	42/34	NS
Age, years	67 ± 13.7	67 ± 11.9	NS
Etiology of liver cirrhosis			
Hepatitis B virus	12 (12)	15 (20)	
Hepatitis C virus	60 (61)	32 (42)	
Alcohol	11 (11)	12 (16)	NS
Nonalcoholic steatohepatitis	5 (5)	5 (7)	
Other	11 (11)	12 (16)	
Liver function (Child-Pugh)			
Grade A	46 (46)	43 (57)	
Grade B	46 (46)	28 (37)	NS
Grade C	7 (7)	5 (7)	

Data given as total number (percentage) or mean ± standard deviation, unless otherwise specified.

SB2: second-generation PillCam SB2; SB3: third-generation PillCam SB3; NS: not significant.

**Table 2:** Diagnostic yield of capsule endoscopy for esophageal varices.

Endoscopic findings	Device		*P* value
	SB2	SB3	
Form			
F1	52% (16/31)	81% (30/37)	<0.05
F2/3	83% (20/24)	81% (17/21)	NS
Location			
Ls/Lm	81% (21/26)	79% (31/39)	NS
Li	52% (15/29)	84% (16/19)	NS
Red color sign (+)	33% (7/21)	65% (13/20)	NS

Esophageal transit time			
<5 seconds	56% (19/34)	72% (18/25)	NS
>5 seconds	81% (17/21)	79% (26/33)	NS

The varices were graded based on esophagogastroduodenoscopy findings as follows: F0, lesion without varicose appearance; F1, straight, small-caliber varices; F2, moderately enlarged, beady varices; F3, markedly enlarged, nodular, or tumor-shaped varices.

SB2: second-generation PillCam SB2; SB3: third-generation PillCam SB3; NS: not significant; Ls: locus superior; Lm: locus medialis; Li: locus inferior.

**(a):** 

SB2 findings	EGD findings		Total
	+	−	
+	36	0	36
−	19	44	63

Total	55	44	99

*κ* value, 0.63; sensitivity, 65%; specificity, 100%; positive predictive value, 100%; negative predictive value, 70%.

**(b):** 

SB3 findings	EGD findings		Total
	+	−	
+	47	0	47
−	11	18	29

Total	58	18	76

*κ* value, 0.67; sensitivity, 81%; specificity, 100%; positive predictive value, 100%; negative predictive value, 62%.

EGD: esophagogastroduodenoscopy; SB2: second-generation PillCam SB2; SB3: third-generation PillCam SB3.

**(a):** 

SB2 findings	EGD findings		Total
	+	−	
+	16	0	16
−	15	44	59

Total	55	44	99

*κ* value, 0.56; sensitivity, 52%; specificity, 100%; positive predictive value, 100%; negative predictive value, 75%.

**(b):** 

SB3 findings	EGD findings		Total
	+	−	
+	30	0	30
−	7	18	25

Total	37	18	55

*κ* value, 0.74; sensitivity, 81%; specificity, 100%; positive predictive value, 100%; negative predictive value, 72%.

EGD: esophagogastroduodenoscopy; SB2: second-generation PillCam SB2; SB3: third-generation PillCam SB3.
